# Manufacturing of Fluff Pulp Using Different Pulp Sources and Bentonite on an Industrial Scale for Absorbent Hygienic Products

**DOI:** 10.3390/molecules27155022

**Published:** 2022-08-07

**Authors:** Saeed Ismaeilimoghadam, Mehdi Sheikh, Pouyan Taheri, Sadegh Maleki, Hossien Resalati, Mehdi Jonoobi, Bahareh Azimi, Serena Danti

**Affiliations:** 1 Department of Wood and Paper Science and Technology, Faculty of Natural Resources, University of Tehran, Karaj 77871-31587, Iran; 2 Marinasun Company, Eshtehard 31881-16881, Iran; 3 Department of Wood and Paper Sciences, Faculty of Natural Resources, Tarbiat Modares University, Nour 46414-356, Iran; 4 Department of Wood and Paper Science and Technology, Agricultural and Natural Resources University of Sari, Sari 48181-66996, Iran; 5 Department of Civil and Industrial Engineering, University of Pisa, 56122 Pisa, Italy

**Keywords:** bagasse, hardwood and softwood pulps, cellulose, baby diaper, sanitary napkin

## Abstract

In this study, for the first time, a composite fluff pulp was produced based on the combination of softwood (i.e., long-length fiber), hardwood (i.e., short-length fiber), non-wooden pulps (i.e., bagasse) and bentonite, with specific amounts to be used in hygienic pads (e.g., baby diapers and sanitary napkins). After the defibration process, the manufactured fluff pulp was placed as an absorbent mass in diapers and sanitary napkins. Therefore, tests related to the fluff pulp, such as grammage, thickness, density, ash content, humidity percentage, pH and brightness, tests related to the manufactured baby diapers, such as absorption capacity, retention rate, retention capacity, absorption time and rewet, and tests related to the sanitary napkin, such as absorption capacity and rewet, were performed according to the related standards. The results demonstrated that increasing the amount of bagasse pulp led to increasing the ash content, pH and density of fluff pulp and decreasing the brightness. The addition of bentonite as a filler also increased ash content and pH of fluff pulp. The results also demonstrated that increasing of bagasse pulp up to 30% in combination with softwood pulp led to increasing absorption capacity, retention rate, retention capacity, absorption time and rewet of baby diapers and of sanitary napkins.

## 1. Introduction

More than 15 different materials are used to manufacture one baby diaper. A polypropylene top sheet that is in direct contact with the baby’s skin and is responsible for receiving fluids, an acquisition distribution layer (ADL), which is mostly colored and its responsibility is to distribute fluids into the diaper, the absorption mass, which is a mixture of fluff pulp and super absorbent polymer (SAP), a back-sheet layer, which is mostly a breathable type and made of polyethylene and its responsibility is to prevent leakage of fluids and, finally, a fastening system, such as tape, frontal tape, glue and others [1]. Fluff pulp, which forms approximately 40% of the total weight of classic diapers, leads to increased absorption speed in diapers and also contributes to the absorption process [2]. In sanitary napkins, fluff pulp forms more than 70% of the total weight of a pad and its responsibility is to distribute the liquids along the length of the pad. Therefore, fluff pulp is one of the most important and strategic materials in the above-mentioned productions. On the other hand, fluff pulp is important in terms of some aspects, such as supplying, transportation, warehousing and logistics. A desirable fluff pulp should provide some specific properties, including appropriate bulk properties, high absorption capacity, low absorption time, high tensile strength and high brightness [2,3]. The fiber length is another important property in the fluff pulp. The higher the fiber length, the higher SAP attraction, and, consequently, the higher the water absorption.

In general, each country produces fluff pulp according to its resources. The United States, for example, uses southern pines to produce fluff pulp. In other countries, such as Iran, among the poorest countries in terms of wood and forest resources, the production of fluff pulp is inevitably limited to the use of imported pulps and non-wood sources, such as bagasse pulp, which is produced in southwestern Iran due to the abundance of sugarcane pulp [4,5]. Non-wood pulps mainly contain short fibers [6], high hemicellulose [4], heavy metals and high ash [4]. The application of pulps containing non-wood fiber in fluff pulp leads to the formation of fluff pulp with high density and short-length fiber, which probably results in unsuitable defibration into the hammermill during the manufacturing of baby diapers and sanitary napkins. On the other hand, ash and minerals, which are mainly present in this type of pulp, lead to the abrasion of hammermill blades [7]. Therefore, the combination of softwood pulp with long-length fibers with pulps made of non-wood fibers, such as bagasse, could be a promising strategy for manufacturing fluff pulp in countries that have limited wood and forest resources.

SAP acts as the main material for absorbing fluids in baby diapers and sanitary napkins and fluff pulp has three main tasks: (i) it aids water absorption to some extent; (ii) carries a large amount of SAP; and (iii) increases the absorption speed in the pads. The absorption capacity of fluff pulp could be increased using some strategies. Among others, a few studies have used minerals and non-wood pulps in order to manufacture fluff pulp. In fact, the addition of minerals, such as bentonite, into the fluff pulp can increase the absorption capacity of fluff pulp due to its superabsorbent properties [8]. Xu et al. (2017), investigated the effect of calcium nano-carbonate on the manufacturing of fluff pulp. They stated that the aim of their research was to improve the absorption capacity of fluff pulp by reducing the hydrogen bond between the fibers due to adding calcium nano-carbonate. Thus, the absorption capacity and the ash content of fluff pulp were increased due to the presence of calcium nano-carbonate, while the burst resistance was decreased [2]. Ebrahimpour Kasmani and Samariha (2021) investigated the effect of montmorillonite nano clay on the properties of chemo-mechanical pulping paper. The result of their study demonstrated that with increasing nano clay up to 2 w%, the tensile strength of the manufactured paper increased, whereas using 10% of it led to a tensile-strength reduction. In addition, the brightness of the manufactured paper decreased with increasing nano clay [9]. Shanmugasundaram and Gowda (2010) investigated the effect of four different fibrous compositions, including pure bamboo, pure organic cotton, bamboo/organic cotton (70/30, *w*/*w*) and bamboo/organic cotton (50/50, *w*/*w*), on the water absorption and rewet of baby diapers. The result demonstrated that the diaper made of bamboo/organic cotton (70/30, *w*/*w*) exhibited the best performance, so that the maximum absorption capacity and the minimum rewet were obtained [10]. 

The purpose of this study was the production of a fluff pulp with specific properties using a combination of different amounts of bagasse pulp, softwood and hardwood pulps for cellulose hygienic (i.e., baby diaper and sanitary napkin) applications. This research was conducted on an industrial scale and the effect of bentonite on the properties of fluff pulp were also investigated. First, fluff pulp was manufactured in a paper factory with different formulations and then it was transferred to a factory that produces baby diapers and sanitary napkins, and finally, the properties of diapers and sanitary napkins obtained from these kinds of fluff pulps were investigated. The application of non-wood sources, such as bagasse pulp, in the combination of fluff pulp on an industrial scale would be promising for the production of products, such as diapers and sanitary napkins, in countries with limited wood sources.

## 2. Results

### 2.1. Manufactured Fluff Pulp 

#### 2.1.1. Morphological Properties

**Figure 1:**
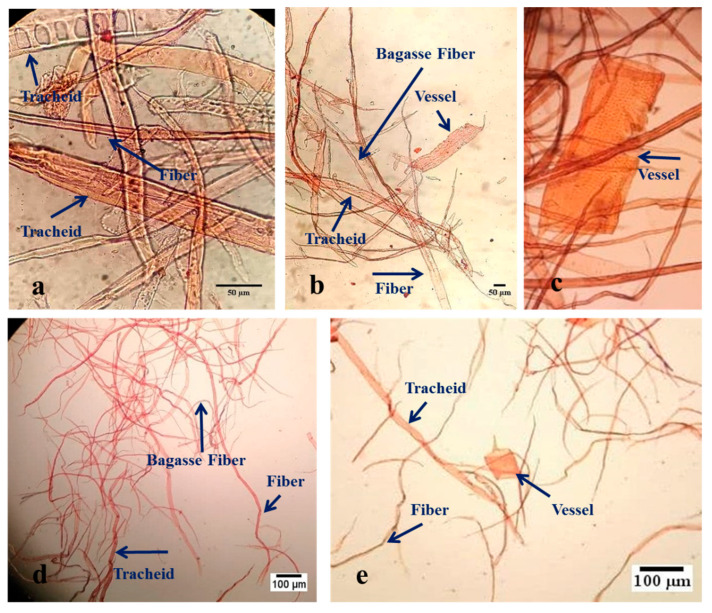
Manufactured fluff pulp with different formulations, 70S30H (**a**,**e**), 50S15H35B (**b**,**d**), 50S50H (**c**).

The results of optical microscopy investigations are shown in Figure 1. As expected, there are two types of cells in the fluff pulp in the formulation of 70S30H (Figure 1a,e). One of these cells is tracheid, which is found only in softwood pulp, and the other one is fiber, which is the main cell in hardwood pulps. Figure 1b,d shows the three components of fluff pulp (50S15H35B). Bagasse fiber also exists in this formulation, which is quite distinct due to its low thickness. In the formulation of 50S50H (Figure 1c), it is expected that another type of cell, such as vessels, exists in the fluff pulp.

**Figure 2:**
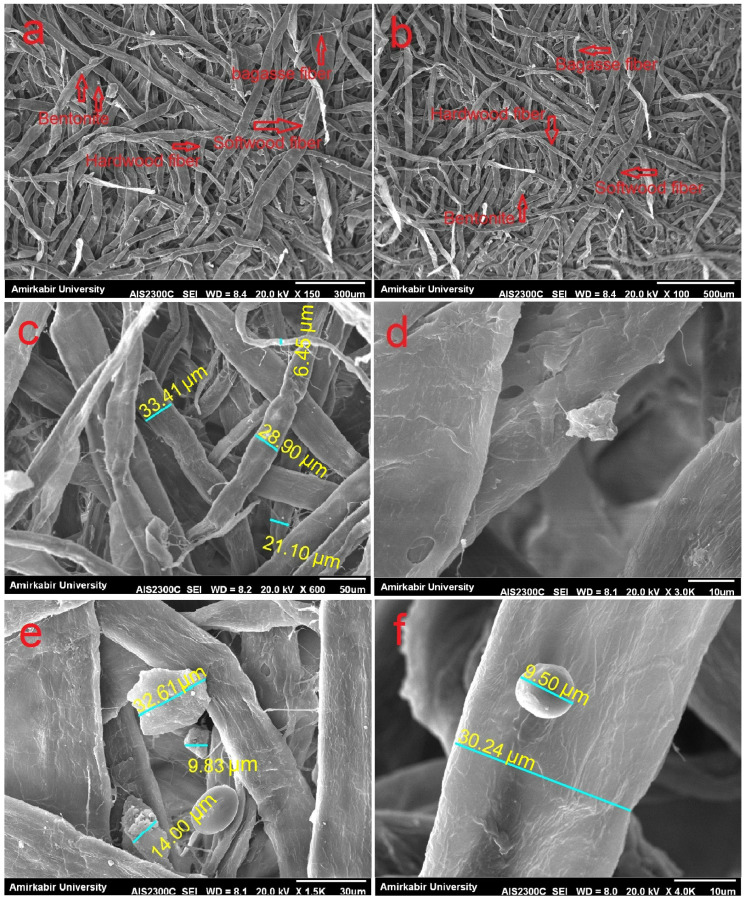
SEM images of fluff pulp with 50S15H35B formulations, distribution of softwood pulp, hardwood pulp and bagasse pulp (**a**,**b**), fiber sizes (**c**) and presence of bentonite on the surface of fibers (**d**–**f**).

SEM images of fluff pulp with 50S15H35B formulations are shown in Figure 2a,b. The uniform combination of all three components, softwood pulp, hardwood pulp and bagasse pulp, was evident in the fluff pulp. Figure 2c demonstrates the diameter of the fibers. In this figure, the presence of softwood fibers, with an average diameter of 30 μm, hardwood pulp with an average diameter of 20 μm and bagasse fibers with an average diameter of 10 μm, is observed. Figure 2d–f shows the presence of bentonite particles on the surface of fibers with uniform distribution. The bentonite particle size located on the surface of fibers ranged from 9 to 32 μm.

#### 2.1.2. Chemical Structure

**Figure 3:**
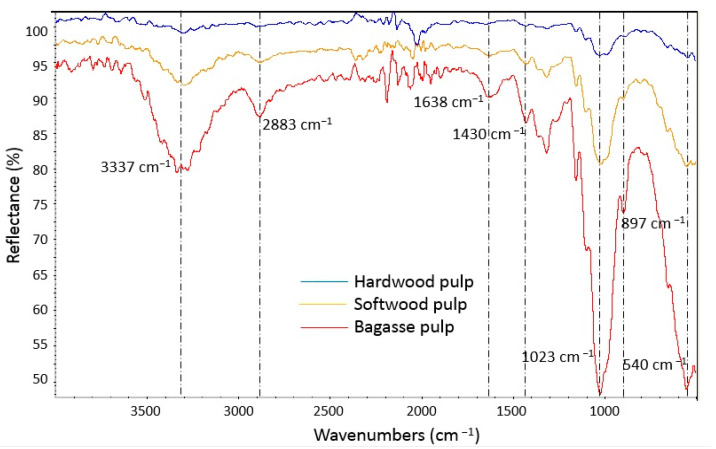
FTIR spectra of softwood pulp, hardwood pulp and bagasse pulp.

FTIR spectra of softwood, hardwood and bagasse pulps are reported in Figure 3. In the spectrum of bagasse and softwood pulps, a broad peak at around 3200–3400 cm^−1^ is assigned to the stretching vibrations of the hydroxyl (–OH) groups of cellulose fibers. The peak at 1638 cm^−1^ refers to the carboxyl groups. The peak at 2880–2890 cm^−1^ is related to the CH stretching vibrations of carbohydrates in polysaccharides. The peak at 1430 cm^−1^ is associated to the CH_2_ symmetric bending, which is related to the main structure of cellulose, while the peak at 897 cm^−1^ is assigned to the glycoside bonds in cellulose and hemicellulose (such as *O*-acetyl-4-*O*-methylglucuronoxylane). The peak at 1020 cm^−1^ is related to the Si-O stretching vibration, whereas that at 540–560 cm^−1^ is related to the Si-O-Si bending vibration.

#### 2.1.3. Physical Properties

**Figure 4:**
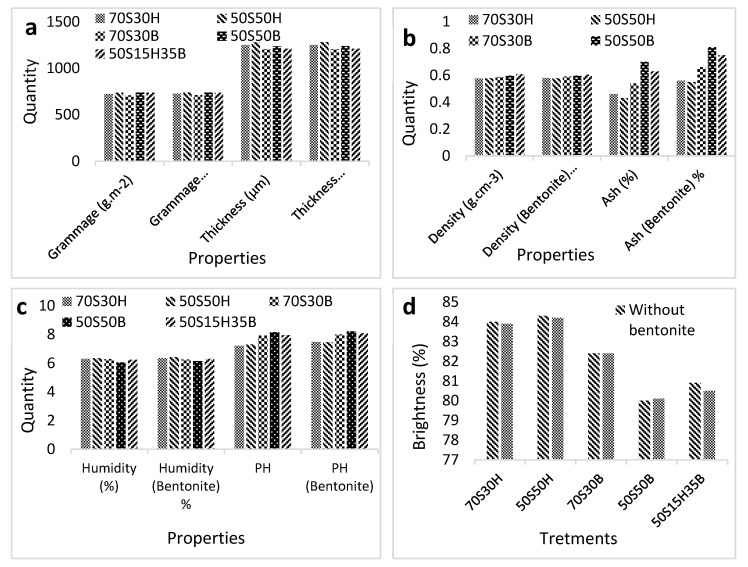
Grammage and thickness (**a**), density and ash content (**b**), humidity and pH (**c**) and brightness (**d**) of manufactured fluff pulp.

Figure 4a reports the grammage and thickness of the fluff pulps produced with different formulations. The results showed that the grammage and the thickness of the manufactured fluff pulps were within the acceptable range (grammage: 700–800 g/m^2^) and (thickness: 1200–1300 μm) in any applied formulations. Based on this figure, there are tiny differences in terms of grammage and thickness of fluff pulps produced with different formulations, while the addition of bentonite (0.1 wt.%) slightly increased the grammage of the fluff pulp, but the addition of bentonite did not cause a significant change in the thickness of fluff pulp. Density and ash content in the different manufactured fluff pulps are reported in Figure 4b. As can be seen, the density of fluff pulp increased in formulations containing bagasse pulp. The addition of bentonite did not cause a significant change in the density of fluff pulp. Increasing the bagasse pulp ratio in fluff pulp led to an increase in the ash content and the addition of bentonite exacerbated this issue. The density of fluff pulp is in the acceptable range (0.57 to 0.63 g/cm^3^) and the ash content is in the appropriate range (less than 1% by weight). The results of humidity percentage and pH of the manufactured fluff pulp revealed no significant difference between the humidity percentages of fluff pulp in different formulations (Figure 4c). The presence of bentonite in fluff pulp also did not have a considerable effect on the humidity percentages of the fluff pulp. The presence of bagasse pulp and bentonite in the fluff pulp led to increasing pH. On the other hand, bentonite, due to its mineral origin, slightly increased the pH in fluff pulp. The moisture content in the manufactured fluff pulp in all formulations was in the acceptable range (5% to 8%) but its pH was slightly higher than the acceptable level (6.5 to 7.5). Figure 4d shows the brightness of fluff pulp in different formulations. According to this figure, increasing the bagasse pulp resulted in a reduction in the fluff pulp brightness. Bagasse pulp has a brightness of about 78% (it is a semi-bleached pulp), while the brightness of imported softwood pulp and hardwood pulp is 85% and 84%, respectively. Therefore, the presence of bagasse pulp reduced the brightness of fluff pulp. The addition of bentonite did not notably change the brightness of the fluff pulp. The acceptable brightness of fluff pulp for hygienic purposes should not be less than 80%, according to the mentioned standard.

### 2.2. Baby Diapers

#### 2.2.1. Absorption Capacity and Retention Rate

**Figure 5:**
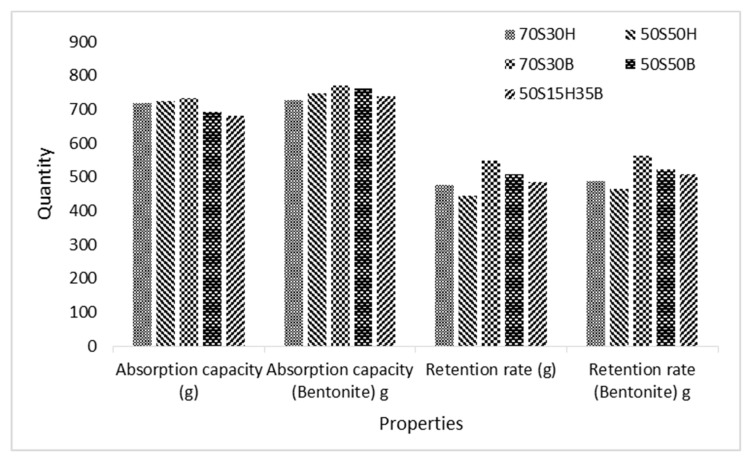
Absorption capacity and retention rate in baby diapers produced with different fluff pulps.

Figure 5 shows the absorption capacity and retention rate of baby diapers manufactured from fluff pulp with different formulations. As can be seen, the highest absorption capacity of diapers (734 g) is related to 70S30B fluff pulp, including 70% softwood pulp and 30% bagasse pulp, approximately 10% higher than the acceptable amount (660 g). The lowest absorption capacity of diapers (660 g) is related to fluff pulp with the formulation of 50% softwood pulp, 15% hardwood pulp and 35% bagasse pulp, about 3% higher than its minimum acceptable amount.

**Figure 6:**
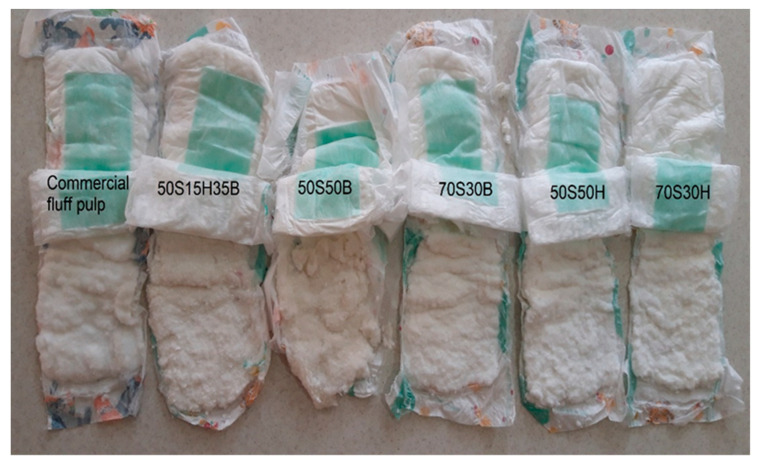
Defibration type of fluff pulp in the baby diapers.

On the other hand, the presence of bentonite as a superabsorbent material in fluff pulp increased the absorption capacity in the diapers. Different defibration forms of fluff pulp in the hammermill are shown in Figure 6. The retention rate in diapers manufactured from fluff pulp with the formulation of 70S30B fluff pulp is higher than the other formulations and the lowest retention rate is for diapers with the formulation of 50S50H fluff pulp. The minimum acceptable retention rate in diapers (size 5) is 410 g.

#### 2.2.2. Retention Capacity, Absorption Time and Rewet

**Figure 7:**
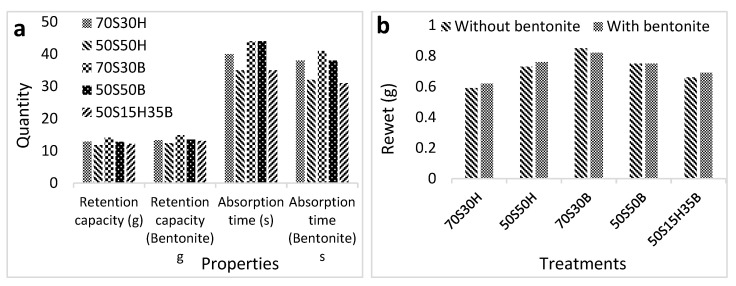
Retention capacity and absorption time (**a**), as well as rewet (**b**) of baby diapers produced with different fluff pulp formulations.

The retention capacity and absorption time of diapers manufactured from fluff pulp with different formulations are shown in Figure 7a. The highest retention capacity is related to diapers manufactured from 70S30B fluff pulp, which was slightly increased by the addition of bentonite. The minimum acceptable retention capacity for diapers (size 5) is 11 gr. The maximum acceptable absorption time in a diaper is 60 s. The results of rewet of baby diapers manufactured from fluff pulp with different formulations are demonstrated in Figure 7b. The lowest rewet is related to diapers manufactured from 70S30H fluff pulp and the highest one is related to diapers made from 70S30B fluff pulp. The maximum allowable amount, again, in baby diapers is 1 g.

### 2.3. Sanitary Napkin

#### Absorption Capacity and Rewet

**Figure 8:**
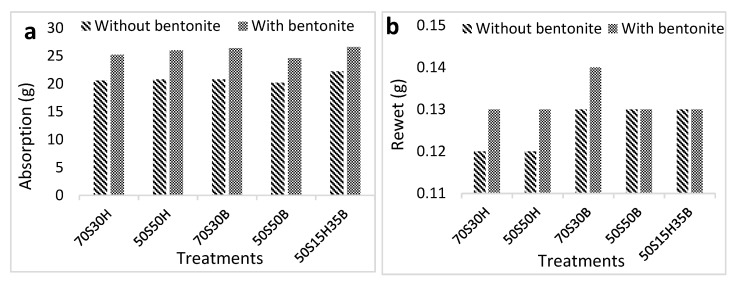
Absorption capacity (**a**) and rewet (**b**) of sanitary napkins produced with different fluff pulp formulations.

**Figure 9:**
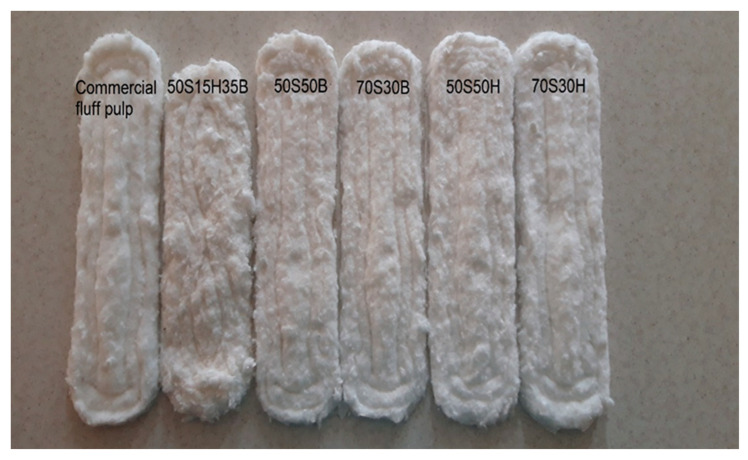
Defibrating types of fluff pulp in the sanitary napkins.

Figure 8a shows the adsorption capacity in the sanitary napkin manufactured from fluff pulp with different formulations. The absorption capacity of all sanitary napkins is higher than the minimum acceptable amount (20 g), but there is no significant difference between the different formulations. The addition of bentonite to fluff pulp (0.1% w%) increased the absorption capacity of sanitary napkins from 16 to 21% in various formulations. As reflected in Figure 8b, there is a tiny difference between the rewet of sanitary napkins manufactured from fluff pulp with different formulations. The addition of bentonite increased the rewet, which is probably because of an increase in water absorption in the sanitary napkin. The maximum acceptable rewet in the sanitary napkin is 1 g. Different defibration forms of fluff pulp in the hammermill are shown in Figure 9.

## 3. Discussion

Absorbent hygienic products (AHPs) are designed to receive, absorb and retain body fluids and solid wastage. The global market for such products has been growing at a significant rate in pace with single-use products. Baby diapers and sanitary napkins are two main products of AHPs, which are considered single-use items [11]. The usage of baby diapers globally highlights that a typical child will use approximately 4100 disposable diapers a year [12]. The absorption mass, which is a mixture of fluff pulp and super-absorbent polymer (SAP), forms more than 70% and 80% of the weight of a diaper and sanitary napkin, respectively. Countries with limited wood and forest resources are faced with some problems in the production of fluff pulp. Indeed, the applied non-wood sources in such countries lead to the formation of fluff pulp with high density and short-length fiber. Our study focused on the development of fluff pulp composed of softwood pulp with long-length fibers, pulps made of non-wood fibers, such as bagasse and hardwood pulps, as a novel strategy to produce cellulose hygienic products in poor countries, in terms of wood and forest resources.

According to the SEM and optical microscopy investigations, the average diameter of the softwood fibers, mainly composed of tracheid, hardwood fibers, mainly composed of fibers and bagasse fibers, was about 30 μm, 20 μm and 10 μm, respectively. Chinga-Carrasco et al., 2011 reported that the length and diameter of the fibers of Eucalyptus, a hardwood species, are 640 μm and 18.8 μm, respectively, and that the length and diameter of pine, a softwood species, are 2070 μm and 34.2 μm, respectively [13]. Hassan et al. 2012, in their research on bagasse fiber, reported that the diameter of bagasse fiber ranged from 5 to 15 μm [14]. Moreover, according to SEM investigation, softwood, hardwood and bagasse pulps were uniformly distributed in fluff pulp. During the production process, the particle size of bentonite located on the surface of fibers was reduced with respect to the ones used in this research (75 µm). This may be due to the mixing process in the pulper.

According to the FTIR spectroscopy, the peak intensity of hydroxyl group [15,16,17] in bagasse pulp is higher than the softwood and hardwood pulps. Because of the chemical compositions of bagasse pulp, which are mainly hydrophilic and contain hydroxyl groups, it was expected that the peak intensity of the hydroxyl group in this pulp was very high. Bagasse pulp is a semi-bleached pulp, meaning that the amount of hemicellulose and lignin in this pulp is higher than the softwood and hardwood pulps. Therefore, it can be expected that the amount of hydroxyl groups is higher in bagasse pulp due to the higher amount of hemicellulose. On the other hand, the crystalline region of cellulose in bagasse pulp is much lower than the other two [18,19]. The peak related to carboxyl groups [20] was found only in bagasse pulp. As previously mentioned, bagasse pulp contains a large amount of hemicellulose, such as Glucuronoxylan, which contains many carboxyl groups [21]. The peak intensity, which is related to the CH stretching vibrations of carbohydrates polysaccharides [15,16,17], in bagasse pulp, was higher than that of the other two types, which is most likely due to the high hemicellulose content in bagasse pulp. The peak intensity related to the CH_2_ symmetric bending and glycoside bonds in cellulose and hemicellulose [16,17,18,19,20,21,22] in bagasse pulp was high, maybe because of a high amount of hemicellulose, such as Glucuronoxylan, in bagasse pulp. The stretching vibration of Si-O and the bending vibration of Si-O-Si [16] in bagasse pulp was more intense. The high ash content of bagasse pulp confirms this.

The results demonstrated that the density of fluff pulp increased in the formulations containing bagasse pulp. It can be expected that in these formulations, the number of hydrogen bonds between the fibers increased since the bagasse pulp contains many short fibers and a large amount of hemicellulose [4]. Increasing the number of hydrogen bonds results in the production of pulp with less bulk and thickness; therefore, considering a constant grammage, it can be expected that the addition of bagasse pulp increases the density of fluff pulp [23]. In general, the density of fluff pulp should not be different from its standard amount. Very-high-density fluff pulp reduces the fiber length after the defibrating process in the hammermill, resulting in a reduction in the adsorption capacity and retention capacity of baby diaper pads. Very low density, on the other hand, causes aggregation (knot), which, again, reduces the adsorption capacity of diapers [24,25].

The ash content in the fluff pulp increased in the formulations containing bagasse pulp and bentonite. Indeed, bagasse pulp contains heavy metals that have a large effect on the ash content in fluff pulp [4,5,6,7,8,9,10,11,12,13,14,15,16,17,18,19,20,21,22,23,24,25,26]. On the other hand, it contains considerable amounts of parenchymal cells that are places of accumulation for minerals, such as silica and silicate [4]. The result of FTIR spectra also showed that the peak related to the minerals is very intensive in the bagasse pulp spectrum. Bentonite also contains mineral compounds, such as sodium, aluminum, magnesium, silicate, etc., which can increase the amount of ash in the fluff pulp [27].

Increasing the bagasse pulp ratio in the fluff pulp led to an increase in pH. Since bagasse pulp is a semi-bleached pulp that is bleached with sodium hypochlorite, it can be expected to have a higher pH than imported soft and hard pulp. The pH can be controlled in the production line by adding a little sulfuric acid.

A remarkable amount of softwood pulp, the presence of bagasse pulp, large amounts of hemicellulose and suitable defibration in the hammermill, caused the water absorption of diapers obtained from 70S30B to be higher than other formulations. The higher water absorption capacity of baby diapers with a higher amount of softwood pulp (70S30B) could be related to the fact that softwood pulp has the longest fiber length [24,25,26,27,28,29], the highest cellulose content [29] and the highest water absorption and retention capacities [30]. Longer fibers form a network with higher porosity [3,4,5,6,7,8,9,10,11,12,13,14,15,16,17,18,19,20,21,22,23,24], which has a greater effect on water absorption than hydrophilicity [31]. On the other hand, based on FTIR results, the carboxyl group peak corresponding to water absorbency is present only in bagasse pulp (due to more hemicellulose, such as Glucuronoxylan, in bagasse pulp) and also the peak intensity of the hydroxyl group in bagasse pulp is higher than softwood and hardwood pulps. Therefore, it is expected that its water absorption capacity will increase with an increase in bagasse pulp percentage in the fluff pulp. However, the highest adsorption capacity of baby diapers was not related to 50S50B, but to 70S30B due to the inappropriate defibrating process of 50S50B formulation in the hammermill, which shows the importance of the type of defibrating process on water absorption compared to the hydrophilicity of its ingredients [31]. It is worth mentioning that although SAP plays the most important role in the absorption capacity of diapers [3], the type of fluff pulp also has a considerable effect on it [3]. The results of absorption capacity also showed that the presence of bentonite led to an increased absorption capacity in diapers. Bentonite is a cationic material able to bond with cellulose fibers with an anionic surface charge [27]. The hydrophilicity of bentonite [32] leads to the formation of strong hydrogen bonds between the hydroxyl groups in the silicate layers of bentonite and the hydroxyl groups of cellulose fibers [33]. These hydrogen bonds are broken into hydroxyl groups through water adsorption by bentonite and cellulose, leading to the increased water absorption capacity of diapers.

The maximum and minimum retention rates in baby diapers were related to the baby diaper manufactured with 70S30B and 50S50H fluff pulp formulations, respectively. In general, the type of fiber in softwood and hardwood pulps is different. Softwood pulps contain tracheid cells and hardwood pulps contain fiber and vessel cells [34]. In fiber and vessel cells, the mechanism of water transfer occurs through cell openings, while water transfer in tracheid cells occurs through punctuations in the cell wall. Thus, water absorption in these cells (tracheid) is as difficult as the process of losing water from fibers. Therefore, the high retention rate of diapers manufactured from 70S30B fluff pulp is due to the presence of 70% softwood pulp containing tracheid cells and the low retention rate of diapers manufactured from 50S50H fluff pulp is due to the presence of 50% of softwood pulp and 50% hardwood pulp, with fiber cells and vessels. Adding bentonite to the formulations led to an increase in retention rate for baby diapers. Bentonite contains silicate layers that, depending on the amount used in the paper, form different structures, such as exfoliation or intercalation [9]. Water molecules probably need to go through a long and tortuous process of bentonite silicate layers to be adsorbed or desorbed. Noteworthily, during the adsorption test, which takes about 30 min, the water molecules had the opportunity to travel this path, while during desorption, which occurs in the centrifuge for 2 min, the water molecules did not have enough time to exit from long and tortuous silicate layers. Therefore, it can be expected that adding bentonite to fluff pulp will increase the retention rate in diapers.

Baby diapers manufactured from fluff pulp with the formulations of 70S30B had a maximum absorption time, while the minimum absorption time was related to the baby diaper manufactured by 50S50H fluff pulp formulations. Diapers manufactured from 70S30B fluff pulp absorbed water slowly, while the ones manufactured from 50S50H fluff pulp absorbed water quickly. Summarizing, by increasing softwood pulp/hardwood pulp ratio in the composition, the absorption time of diapers increased. As mentioned above, softwood pulp contains tracheid cells that absorb water with difficulty and over a longer period of time, while hardwood pulp containing fiber and vessel cells absorb water easily and in a shorter time.

The maximum and minimum rewet in baby diapers was related to the baby diaper manufactured with 70S30B and 70S30H fluff pulp formulations, respectively. When fibers absorb water, the capillary nature of the fibers gradually decreases, which, in turn, means the water absorption rate in the fibers is decreased. Due to water absorption, fiber strength decreases. Therefore, the more water absorption by the fibers, the more resistance reduction. Thus, fibers that absorb a lot of water lose more water under pressure [3,4,5,6,7,8,9,10,11,12,13,14,15,16,17,18,19,20,21,22,23,24,25,26,27,28,29,30,31].

There is a little difference between the absorption capacity of sanitary napkins manufactured from different fluff pulp formulations. The mechanism of water absorption in diapers and sanitary pads is different, so that, in diapers, actual absorption occurs, while in sanitary napkins, the absorption is of a diffusion type [35]. It can be hypothesized that the absorption capacity in the sanitary napkin largely depends on the type of dry-defibration process in the hammermill. In this research, since the dry defibrating of most of the fluff pulp was not suitable, especially in fluff pulp containing bagasse pulp, it was expected that the absorption capacity is as low as possible. The addition of bentonite led to increasing the absorption capacity, which is probably due to the absorbent nature of bentonite, as well as the high retention of bentonite particles, which overall, prevented water from spreading to the sides of the sanitary napkin.

The search for better performing, ecologically sustainable, bio-based and biodegradable or compostable diapers is one of the high-impact materials challenges [36]. For this purpose, modified polysaccharides forming hydrogels, such as those derived from mushrooms or algae [37,38], in combination with fluff pulp and SAP, may offer great opportunities in improving the expected sustainability goals.

## 4. Materials and Methods

### 4.1. Materials

In this research, softwood pulp with an average fiber length of 2.5 mm, an average fiber diameter of 30 μm and brightness of 88–92% was supplied from SCA Co. (Sundsvall, Sweden). Hardwood pulp with an average fiber length of 0.84 mm, an average fiber diameter of 20 μm and a brightness of 90% was supplied from Bracell Co. (Sao Paulo, Brazil). Bagasse pulp with an average fiber length of 0.7 mm, an average fiber diameter of 10 μm and brightness of 78% was supplied from Pars Co. (Ahvaz, Iran). Sodium chloride and safranin were purchased from Sigma Aldrich (St. Louis, MO, USA) and Bentonite with 200 mesh size and containing 86% aluminosilicate (75.5% silicate and 10.5% aluminum) was supplied from Pak Bolur Narin Co. (Semnan, Iran).

### 4.2. Fluff Pulp Manufacturing

First, softwood pulp, hardwood pulp and bagasse pulp with specific weight (Table 1) were transferred into an industrial pulper in Ariyo Cellulose Factory in the north of Iran and were completely mixed for 20 min with a consistency of 3 w/v% until the fibers were completely separated. Subsequently, the formulations in Table 1 were again manufactured at 0.1 w% bentonite (based on the total weight of the pulps and as a replacement for softwood pulp) was added in each formulation.

**Figure 10:**
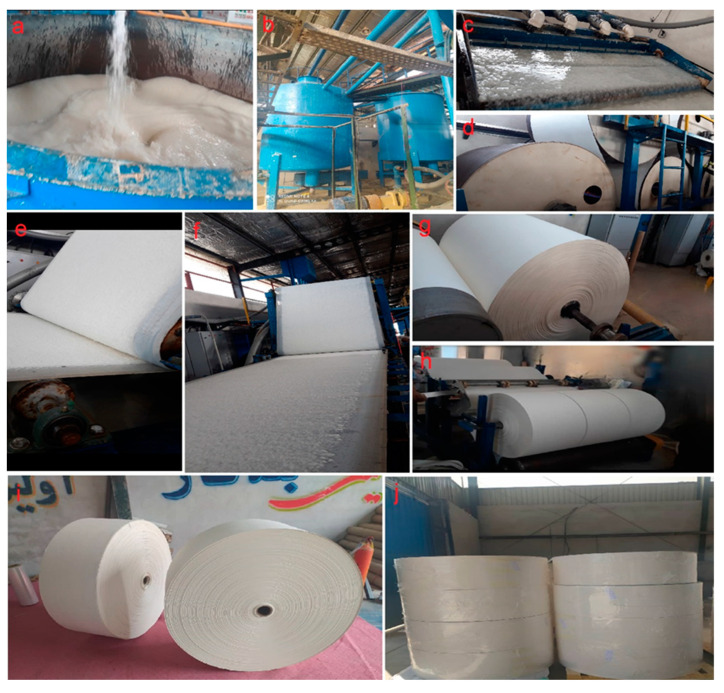
The processing of fluff pulp manufacturing. Pulper (**a**), storage towers (**b**), headbox (**c**), dryers (**d**), top and bottom wires (**e**,**f**), mother roll in the paper machine (**g**), three rolls in rewind machine (**h**) and packed rolls (**i**,**j**).

The process of manufacturing fluff pulp, which was performed at Ariyo Cellulose Factory (Sari, Iran), is shown in Figure 10. After the formation of fiber suspension inside the pulper (Figure 10a), the fluff pulp was pumped into storage towers (Figure 10b). The fiber suspension was then pumped from the storage towers into a wire pit at the bottom of the paper machine where it was diluted with the returned water from the production line and then transferred into the level box. After reaching a certain level in the level box, the suspension was transferred into the head box (Figure 10c) and thereafter, was poured on the wire. The linear speed of the wire with a width of 140 cm was about 12 m/min. Gradually, as the wet pulp was moved toward the dryers (Figure 10d), its moisture content decreased and its consistency increased. Just before press # 1, a strong vacuum drained much water from the pulp to stop the pulp network structure from collapsing under the press. The pulp was placed on the first dryer cylinder after passing through 3 presses and reaching a suitable moisture level. In this production line there were two wires, the lower and upper parts (Figure 10e,f), which carry the pulp, placed on top of each other before press # 1. The production line consisted of 10 dryers, 5 located on the top, responsible for drying the back of the fluff pulp, and 5 located on the ground, responsible for drying the surface of the paper. After passing these dryers, the manufactured pulp was finally collected on a metal core (Figure 10g) and was sent to the rewind machine. The grammage and humidity percentage of the fluff pulp were checked every 30 min.

The final width of the fluff pulp roll (i.e., 132 cm), which was made in the paper machine part, was turned into 3 rolls of fluff pulp with a width of 41 cm and a diameter of 120 cm in rewind machine (Figure 10h). The grammage and humidity percentage of the fluff pulp were 750 ± 25 g/m^2^ and 5% ± 2%, respectively. All machines including pulpers, storage towers, paper machines and rewind machines were made in Iran, Mohammadi Co. (Sari, Iran). After packing (Figure 10i,j), the rolls of fluff pulp were transferred to Marinasun Cellulose Industries Factory (Karaj, Iran), a manufacturer of baby diapers and sanitary napkins.

### 4.3. Chemical and Morphological Characterization of Pulps

Fourier transfer infrared spectroscopy (FTIR), model NICOLET T380 (Thermo Scientific, Waltham, MA, USA), was used for investigating functional groups in softwood pulp, hardwood pulp and bagasse pulp. Scanning electron microscopy (SEM), model EM3200, made in China, KYKY Co, under a voltage of 20 kV, was used for the morphological investigation of manufactured fluff pulp. For this purpose, the samples were dried for 24 h at 103 ± 2 °C and then sputter coated with gold. Optical microscopy, model Eclipse 50i, (Japan) was used for morphological investigation of different cells in the fluff pulp. For this purpose, 1 g fluff pulp from each formulation was placed in a glass tube and 10 mL water was poured into the tube. Then the tube was placed in an oven at 60 °C for 6 h. After this time, 1 mL Safranin (0.5 w/v%) solution was added to the mixture to make the fibers visible under microscope. From each formulation, 2 drops of the prepared mixture were poured on a glass slide and finally the morphological study of fibers was performed using 40× magnification.

### 4.4. Physical Properties of Fluff Pulp

Different properties of fluff pulp were controlled by the input item section of the quality control unit, at Marinasun Cellulose Industries Company.

Grammage of fluff pulp was calculated according to Formula (1) (Iranian National Standard # 471) [39], where G is the grammage of fluff pulp in (g/m^2^), W is the weight of fluff pulp in (g) and A is the area of fluff pulp in (m^2^) (Equation (1)). The acceptable grammage range of fluff pulp according to the standard is 725–775 g/m^2^.

G = W/A(1)

Thickness of fluff pulp was measured by a caliper with an accuracy of 1 µm in accordance with Iranian National Standard # 151 [40]. The acceptable range of thickness of fluff pulp according to the standard is 1100–1300 µm.

Density was calculated according to Equation (2) (Iranian National Standard # 5911) [41], where D is the density in (g/cm^3^), G is the grammage in (g/m^2^) and T is the thickness in (μ). The acceptable density range for fluff pulp according to the standard is 0.5–0.6 g/cm^3^.

D = G/T(2)

Ash content in fluff pulp was calculated according to the Iranian National Standard # 1119 [42]. At first, 1 g of fluff pulp was dried in an oven for 1 h at 103 ± 2 °C. Then, the oven dry weight of the fluff pulp after being placed in the desiccator for 15 min was calculated by a digital scale with an accuracy of 0.001 g. During the drying process of the fluff pulp, one crucible was placed in an oven at a temperature of 550 ± 25 °C. After 1 h, the crucible was placed in a desiccator for 15 min to cool down. The dry weight of the crucible was calculated by a digital scale. The fluff pulp was placed inside the crucible and was placed in an oven at 550 ± 25 °C for 3 h. After this time, the crucible was placed inside the desiccator and weighed again by the scale. The difference between the weight of a crucible without and containing ash determines the weight of the ash. Finally, the ash content was calculated according to Equation (3), where A is the ash content in (%), W_A_ is the weight of ash (g) and W_F_ is the weight of fluff pulp (g). The maximum acceptable ash content for fluff pulp according to the standard is 0.5% and for baby diapers and sanitary napkins is 1.5%.

A = W_A_/W_F_ × 100 (3)

Humidity percentage test was conducted according to the Iranian National Standard # 559 [43]. The 5 g of fluff pulp was weighed by a scale with an accuracy of 0.001 g and then was placed in an oven at 103 ± 2 °C for 2 h. It was then placed in a desiccator for 15 min and the samples were weighed again. Humidity percentage was calculated according to Equation (4), where MC is the humidity content in (%), W_h_ is the weight of the fluff pulp before being placed in the oven (g) and W_o_ is the oven dry weight of the fluff pulp (g). The acceptable humidity content of fluff pulp according to the standard range is 5–8%.

MC = (W_h_ − W_o_)/W_h_ × 100 (4)

pH was measured according to Iranian National Standard # 3568-1 [44]. Briefly, 2 g of fluff pulp was placed in 100 mL of distilled water and the solution was thoroughly stirred in a magnetic stirrer for 30 min. After 30 min, the pH of the solution was measured by a pH meter. The acceptable pH range according to the standard range is 6.5–8.0.

Brightness of fluff pulp was measured according to Iranian National Standard # 13366-2 [45]. One sample in the shape of a circle was prepared and its brightness was measured by a brightness machine (Drick brand, model 2013, made in China). The brightness of fluff pulp according to the standard should not be less than 80%.

### 4.5. Production of Baby Diapers and Sanitary Napkins Using Manufactured Fluff Pulp

Baby diapers from Merci+ brand were produced using GDM machine (General Disposable Machinery, model 2010, Offanengo, Italy) made in Italy. All diapers were produced from size 5, which is suitable for children weighing 12–25 kg, due to the uniformity of their characteristics. This type of manufactured diaper is the classic type in which the amount of defibrated fluff pulp is defined as about 15.5 g, namely about 40% of the total weight of the diaper (39 g), according to the bill of material (BOM).

**Figure 11:**
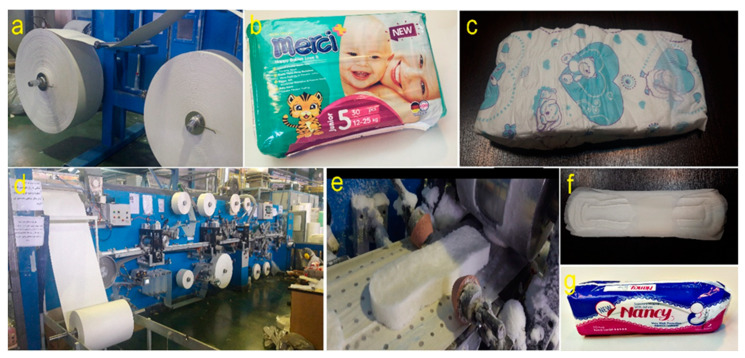
Different stages in production of baby diapers and sanitary napkins. Fluff pulp rolls working on the machine (**a**), baby diaper pack (**b**), baby diaper pad (**c**), BMT machine (**d**), fluff pulp in the shape of sanitary napkin (**e**), sanitary napkin pad (**f**), sanitary napkin pack (**g**).

Sanitary napkins from Nancy brand, in very large size, were produced using BMT machine model 2018 (Behdasht Mashin Tehran, Tehran, Iran) made in Iran. According to BOM, about 8.5 g of fluff pulp was used in each sanitary napkin, which makes up about 70% of its total weight (12.5 g). From each product, 20 pads were randomly selected and tested. Figure 11 shows the different stages of production of baby diapers and sanitary napkins.

### 4.6. Tests Performed on Baby Diapers

After producing the diapers and sanitary napkins, they were evaluated in the laboratory of Marinasun Cellulose Industries Company by the quality control unit, which is mentioned in the following section. Only the tests that are affected by fluff pulp were considered for this study.

**Figure 12:**
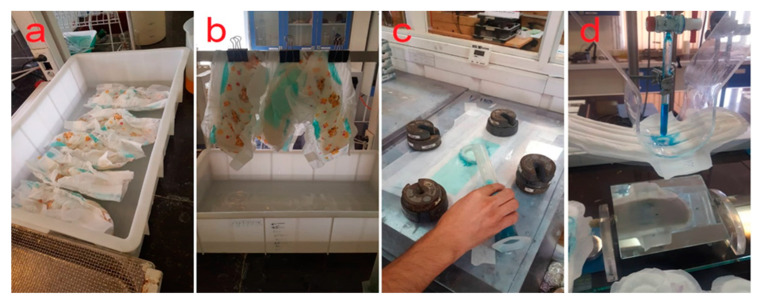
Experimental activity for testing of baby diapers and sanitary napkins. Absorption capacity test of baby diaper pad (**a**,**b**), rewet and absorption time of baby diaper pad (**c**), absorption capacity test of the sanitary napkin pad (**d**).

Absorption capacity was performed in accordance with the modified (i.e., stricter) method of Iranian National Standard, # 14739-1 [46]. The weight of the diaper was first measured by a scale with an accuracy of 0.001 g and then placed in a water tank with a concentration of 0.9% sodium chloride for 30 min (Figure 12a). At this time, the diapers were hung by a metal clip for 5 min (Figure 12b) and weighed again by a scale. The weight difference between the dry and wet states is known as the absorption capacity in grams. According to the standard, the amount of absorption capacity in size 5 diapers should be at least 660 g.

Retention rate and retention capacity were evaluated. After calculating the absorption capacity, the retention rate was sequentially evaluated. The wet diapers were placed in a special centrifuge with 1400 rpm, THOMAS brand, made in Germany, for 2 min and weighed again by the scale. The difference between the diaper mass before and after centrifugation was determined as the retention rate in g. The ratio of retention rate to the initial dry weight is identified as retention capacity in g. These tests were performed in accordance with the Iranian National Standard, # 14739-1 [46]. According to the standard, the retention rate for size 5 diapers is 410 g and the retention capacity for this diaper is 11 g.

Absorption time and rewet tests were performed in accordance with the Iranian National Standard # 3755 [47]. For this purpose, a stretched diaper was located on a plastic plate in which the center of the diaper corresponded to the center of the plate. In the next step, a Plexiglas plate was placed on the diaper and the center of the diaper was aligned with the center of the circle. Proportionate to the size of the diaper (size 5), 11 kg weight (including the weight of the plate) was placed on the Plexiglas plate (Figure 12c) then, according to the size of the diaper, 80 mL of liquid was poured on the diaper in three stages with 20 min intervals. At each stage, the time it took for the liquid to be completely absorbed by the diaper was measured and their sum was recorded as an absorption time. After 60 min, the Plexiglas plate was taken away. Then, three bundles of completely dried paper (m1, m2, m3) were placed in various places: one was placed in the center of the diaper and the other two were placed at the top and bottom of the first batch. In the next step, an object weighing 820 g was placed on each bundle of paper for 10 min. The bundles of paper were then weighed again (n1, n2, n3). The mass difference in each group of papers (R1, R2, R3) was calculated as the bundle (i = 1, 2, 3) weight difference: Ri = (ni - mi). Finally, the rewet (R) was calculated according to Equation (5). The maximum absorption time and the maximum rewet according to the standard are 60 s and 1 g, respectively.

R = (R_1_ + R_2_ + R_3_) (5)

### 4.7. Tests Performed on Sanitary Napkins

Absorption capacity test was performed in accordance with the Iranian National Standard # 1830-1 [48]. At first, a sanitary napkin pad was weighed and then was placed on a device in an arched shape (i.e., to mimic the position created on the pad in the body) (Figure 12d). Then the valve of the device was opened and the blue solution was poured on the pad at a speed of 25 drops per minute. The space between the sanitary napkin pad and the pipe was 3 cm. The test was concluded when the blue solution reached the sides of the pad. After the test, the pad was weighed again and the difference between the weight before and after the test indicated the absorption capacity in the pad. According to the standard, the minimum amount of absorption capacity in a very-large-sized sanitary napkin should not be less than 20 g.

Rewet test was carried out in accordance with the Iranian National Standard # 1830-1 [48]. A sanitary napkin pad was placed on a plastic plate and an inflexible Plexiglas pipe with an inner diameter of 2.4 cm and a height of 5.0 cm was placed on the pad in the center. Thereafter, 7 mL of sodium chlorine solution was poured into the pipe on the pad. After 4 min, a pre-dried and weighed piece of paper was placed on the wet part of the pad and a 3.1 kg weight was placed on it. After 2 min, the papers were weighed again and the rewet amount was calculated using Equation (5). The maximum amount of rewet in a very large size of a sanitary napkin according to the standard should not exceed 1 g.

## 5. Conclusions

In this research, the possibility of manufacturing fluff pulp using long-length fibers (softwood pulp), short-length fibers (hardwood pulp and bagasse pulp) and bentonite was investigated. The pulps were mixed together with different ratios on an industrial scale and were turned into fluff pulp. The result demonstrated that with an increase in bagasse pulp, density, ash content and pH, fluff pulp increased. Fluff pulp with a formulation of 70% softwood pulp and 30% bagasse pulp had the highest absorption capacity, retention rate, retention capacity, absorption time and rewet in baby diapers. The highest rewet in sanitary napkins was related to fluff pulp with a formulation of 70/30 (softwood pulp/bagasse pulp), but the highest absorption capacity in sanitary napkins was related to fluff pulp with a formulation of 50/15/35 (softwood pulp/hardwood pulp/bagasse pulp). The presence of bentonite in fluff pulp also increased the absorption capacity and ash content in the productions. According to the obtained results, we showed that fluff pulp based on softwood pulp, hardwood pulp, bagasse pulp and bentonite is promising in the production of products, such as baby diapers and sanitary napkins, in countries that are deprived of forest resources, such as Iran.

## Figures and Tables

**Table 1:** Percentages of softwood pulp, hardwood pulp and bagasse pulp in different treatments.

Treatment Code	Softwood Pulp (w%)	Hardwood Pulp (w%)	Bagasse Pulp (w%)
70S30H	70	30	0
50S50H	50	50	0
70S30B	70	0	30
50S50B	50	0	50
50S15H35B	50	15	35

S (Softwood pulp), H (Hardwood pulp), B (Bagasse pulp).

## Data Availability

The data presented in this study are available on request from the corresponding authors.

## Sample Availability

Samples of the compounds are available from the authors.
